# Predicting Blood Concentration of Tacrolimus in Patients With Autoimmune Diseases Using Machine Learning Techniques Based on Real-World Evidence

**DOI:** 10.3389/fphar.2021.727245

**Published:** 2021-09-24

**Authors:** Ping Zheng, Ze Yu, Liren Li, Shiting Liu, Yan Lou, Xin Hao, Peng Yu, Ming Lei, Qiaona Qi, Zeyuan Wang, Fei Gao, Yuqing Zhang, Yilei Li

**Affiliations:** ^1^ Department of Pharmacy, Nanfang Hospital, Southern Medical University, Guangzhou, China; ^2^ Beijing Medicinovo Technology Co. Ltd., Beijing, China; ^3^ Dalian Medicinovo Technology Co. Ltd., Dalian, China; ^4^ Zhongshan School of Medicine, SYSU, Guangzhou, China

**Keywords:** blood concentration prediction, therapeutic drug monitoring, machine learning, tacrolimus, autoimmune disease

## Abstract

Tacrolimus is a widely used immunosuppressive drug in patients with autoimmune diseases. It has a narrow therapeutic window, thus requiring therapeutic drug monitoring (TDM) to guide the clinical regimen. This study included 193 cases of tacrolimus TDM data in patients with autoimmune diseases at Southern Medical University Nanfang Hospital from June 7, 2018, to December 31, 2020. The study identified nine important variables for tacrolimus concentration using sequential forward selection, including height, tacrolimus daily dose, other immunosuppressants, low-density lipoprotein cholesterol, mean corpuscular volume, mean corpuscular hemoglobin, white blood cell count, direct bilirubin, and hematocrit. The prediction abilities of 14 models based on regression analysis or machine learning algorithms were compared. Ultimately, a prediction model of tacrolimus concentration was established through eXtreme Gradient Boosting (XGBoost) algorithm with the best predictive ability (*R* ^2^ = 0.54, mean absolute error = 0.25, and root mean square error = 0.33). Then, SHapley Additive exPlanations was used to visually interpret the variable’s impacts on tacrolimus concentration. In conclusion, the XGBoost model for predicting blood concentration of tacrolimus on the basis of real-world evidence has good predictive performance, providing guidance for the adjustment of regimen in clinical practice.

## Introduction

Tacrolimus is a calcineurin inhibitor and widely used immunosuppressive drug in solid organ transplant recipients and patients with autoimmune diseases (e.g., lupus nephritis, systemic lupus erythematosus [SLE], and nephrotic syndrome). (Johnston, 2013; Hannah et al., 2016; Mok, 2017a; Hao et al., 2018) The therapeutic window of tacrolimus is narrow, and inter-individual variability in dose requirement is high. (Johnston, 2013; Andrews et al., 2017; Mok, 2017b) Therapeutic drug monitoring (TDM) is an important approach for personalized medicine of immunosuppressive drugs, which helps to control drug dosage, minimize drug toxicity, guide therapies, and improve patient care. (Cremers et al., 2016; Zhang and Zhang, 2018)

Reasonable plasma tacrolimus concentration can effectively reduce the incidence of adverse drug reactions. According to previous studies, tacrolimus can lead to renal dysfunction, new onset hypertension, and hyperglycemia in patients with lupus nephritis. (Miyasaka et al., 2009; Hannah et al., 2016) In patients with SLE, tacrolimus can result in infections, nephrotoxicity, liver function disorders, nausea, hypertension, anemia, leukopenia, tremors, and itching. (Huang et al., 2019) Furthermore, blood concentrations of tacrolimus appear to be related to acute nephrotoxicity, neurotoxicity, diabetogenicity, and infections in the treatment of lupus nephritis. (Mok, 2017b; Chen et al., 2020) Thus, TDM of tacrolimus is important to reduce adverse effects and ensure adequate drug exposure.

With the rapid development of information technology, the real-world evidence (RWE) derived from medical records has become an important data source for clinical research. RWE is rooted in real clinical practice and comes from a wide range of sources, including electronic medical record examination and imaging data follow-up records during diagnosis and treatment. The research of RWE is a process of data mining, model building, and clinical feature data extraction. Machine learning is suitable for processing a large volume of real-world data, dealing with missing value and high-dimensional data, and capturing complicated relationships between variables, especially for retrospective studies. Recently, some algorithms with more sophisticated principles have been developed, such as eXtreme Gradient Boosting (XGBoost), random forest, K-nearest neighbor (KNN), light gradient boosting machine (LightGBM), and Categorical Boosting (CatBoost), which have been highly recognized in algorithm competitions. (Aha et al., 1991; Breiman, 2001; Chen and Guestrin, 2016; Ke et al., 2017; Prokhorenkova et al., 2017) XGBoost is one of the intelligent classifier construction algorithms, seen in various classification or regression studies with promising prediction results, such as the risk prediction model for type 2 diabetes. (Xu and Wang, 2019) With the increasing number of input subject data, the machine learning model can continually optimize parameters to achieve better accuracy and practicality.

We aimed to identify important influencing variables for tacrolimus concentration on the basis of real-world data and establish a prediction model of tacrolimus concentration in patients with autoimmune disease *via* machine learning techniques to assist clinical regimen decisions.

## Methods

### Study Population

**FIGURE 1:**
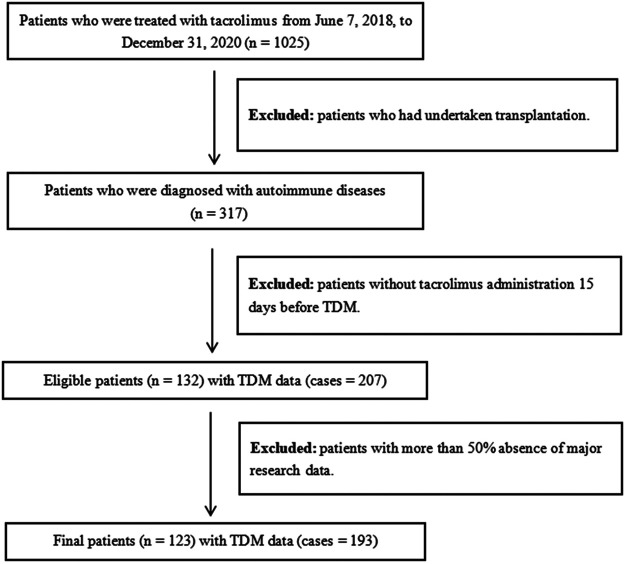
Workflow of patients selection.

We enrolled inpatients who were diagnosed with autoimmune diseases and treated with tacrolimus at Southern Medical University Nanfang Hospital from June 7, 2018, to December 31, 2020. The inclusion criteria included the following: 1) patients who were administered tacrolimus 15 days before TDM; 2) patients who were diagnosed with autoimmune diseases, including lupus nephritis, SLE, and nephrotic syndrome. The exclusion criteria included the following: 1) patients with more than 50% absence of major research data; 2) patients who had undertaken transplantation. The workflow of patient inclusion is illustrated in Figure 1. The final dataset included 123 patients with 193 cases of tacrolimus TDM information, and the final dataset was divided into training and testing groups according to the ratio of 8:2.

Study data have been fully deidentified, and confidential information of patients has been deleted, in accordance with the CIOMS/WHO International Ethical Guidelines for Health-related Research Involving Humans (2016). Consequently, the study was deemed exempt from informed consent by study participants.

### Data Processing

Tacrolimus patient data were extracted from the hospital information system. The interval between two TDM tests of the same patient was mainly distributed within 7–15 days and over 1 month. Hence, samples could be considered independent of each other. We firstly extracted the frequency and dose of tacrolimus ordered by the doctor closest to the TDM test. Drug names (dosage) of tacrolimus included Procofol (0.5 mg*50 tablets), Hongshin (0.5 mg*50 tablets), Serfol (0.5 mg*50 tablets), Neoprocofol (1 mg*50 tablets; sustained-release capsules), and Formexin (1 mg*50 capsules), all in capsule form. Additionally, the last laboratory data before tacrolimus TDM were extracted as essay index, and variables with missing value over 50%, variables completely unrelated to tacrolimus TDM clinically, and classified variables with severe imbalance were deleted. In terms of combination medication, medical order information within 15 days before tacrolimus TDM was extracted, and combination medication was extracted according to drug classification. Given that some drugs did not show in the dataset, the combination medication actually extracted included glucocorticoid (dexamethasone, methylprednisolone, prednisone, cortisone), proton pump inhibitors (PPIs; omeprazole, pantoprazole, ilaprazole, rabeprazole, lansoprazole, ranitidine), calcium channel blockers (CCBs; nifedipine, amlodipine, nitrendipine, felodipine, diltiazem), other immune inhibitors (ciclosporin, mycophenolate mofetil, cyclophosphamide, azathioprine, methotrexate), clarithromycin, and azithromycin. Considering the large amount of missing genetic information, it was not considered in this study.

### Variable Selection

On the basis of tacrolimus patient record data, the relevant important variables were screened from multiple influencing factors. To be specific, we included patient’s demographic information (e.g., gender, age, height, and weight), medication information (e.g., dosage, frequency, and daily dose of tacrolimus), assay index (e.g., liver function index, kidney function index, and routine blood test), and combination medication (e.g., glucocorticoids, PPIs, CCBs, other immune inhibitors, and clarithromycin/azithromycin). The TDM value of tacrolimus was set as the target variable.

Variable selection was performed using sequential forward selection (SFS) algorithm based on XGBoost to select variable subsets with the minimum size and optimum performance. First, data transformation was carried out on continuous variables using logarithm. Then, all 52 variables were selected using XGBoost model with comparison of model performance, which was measured by R-square (*R* ^2^), mean absolute error (MAE), and root mean square error (RMSE). *R* ^2^ is the squared correlation between predicted and actual TDM values (square root of tacrolimus TDM value), with higher value indicating better predictive ability. MAE is the average of the absolute difference between actual and predicted TDM values, and RMSE is the square root of the mean square error between predicted and actual TDM values; hereon, lower values indicate better predictive ability. We examined the XGBoost model performance of the most prominent variable and identified the point at which there was no considerable gain in *R* ^2^ and no considerable loss in MAE and RMSE when adding the next variable to the model. Random forest was used for the interpolation of missing values.

### Model Establishment

Using the selected important variables as covariates, we established and analyzed five linear models (linear regression, LASSO regression, ridge regression, elastic net regression, Bayesian ridge regression) and nine machine learning models (KNN, support vector regression [SVR], random forest, XGBoost, LightGBM, CatBoost, NGBoost, AdaBoost, and GradientBoosting). The prediction performance of all models was evaluated through *R* ^2^, MAE, and RMSE. Ultimately, the model with the highest *R* ^2^ and lowest MAE and RMSE was selected as the final model to predict tacrolimus TDM value. In the testing cohort, the relationship between the predicted value and the true value of tacrolimus TDM value in the final model was obtained.

### Clinical Interpretation

The importance of variables refers to the degree to which each variable in the model contributes to improving the predictive power of the whole model. Herein, we calculated and ranked the importance scores of variables using the algorithm with the best predictive performance. Variables with higher importance scores were more closely related to the accurate prediction of tacrolimus TDM value. Afterward, we used the SHapley Additive exPlanations (SHAP) to visually interpret the impacts of important variables on the model output. (Lundberg and Lee, 2017) Specifically, SHAP could help to explain which variables have positive or negative impacts on predicting tacrolimus TDM value.

## Results

### Baseline Information

A total of 193 cases of tacrolimus TDM data from 123 patients were included in the study. The study population’s baseline information is shown in Table 1. The continuous variables were described by “median (interquartile range, IQR),” while the classified variables were described by “frequency (percentage, %).” The median tacrolimus TDM value was 4 (IQR 3–6) ng/ml. The median dosage of tacrolimus was 1 mg, the median frequency was 2 times/day, and the median daily dose was 2 mg. The median age of cases in this study was 18 (IQR 8–46) years, the median height was 157 (IQR 122–166) cm, the median weight was 53 (IQR 33–65) kg, the median body mass index was 21 (IQR 17–24) kg/m^2^, and the proportion of male patients was 57.51%. The percentage of using combination medication was 85.49% for glucocorticoids, 48.7% for PPIs, 22.28% for CCBs, 17.1% for other immune inhibitors, and 9.33% for clarithromycin/azithromycin.

**TABLE 1:** Variables characteristics.

Categories	Variables		Cases (N = 193)	Missing rate (%)
Target variable	Tacrolimus TDM, ng/ml, median (IQR)		4 (3– 6)	0
	Age, y, median (IQR)		18 (8–46)	0
	Height, kg, median (IQR)		157 (122–166)	3.1
	Weight, cm, median (IQR)		53 (33–65)	0.5
	BMI, kg/m^2^, median (IQR)		21 (17–24)	3.1
	Gender, n (%)	Male	111 (57.5)	0
				Female	82 (42.5)	0
Tacrolimus information	Tacrolimus dosage, mg, median (IQR)		1 (1, 1)	0
	Tacrolimus daily dose, mg, median (IQR)		2 (1, 3)	0
	Tacrolimus using frequency, times/d, median (IQR)		2 (1, 2)	0
Combination	Glucocorticoid, n (%)		165 (85.5)	0
	PPI, n (%)		94 (48.7)	0
	CCB, n (%)		43 (22.3)	0
	Other immune inhibitors, n (%)		33 (17.1)	0
	Clarithromycin/Azithromycin, n (%)		18 (9.3)	0
Essay index	CRP, mg/L, median (IQR)		0 (0–1)	34.7
	ALT, U/L, median (IQR)		15 (10–21)	10.9
	AST, U/L, median (IQR)		16 (13–21)	7.8
	Transaminase ratio, median (IQR)		1 (0–1)	7.3
	PTR, median (IQR)		0 (0–1)	22.8
	BUN, mmol/L, median (IQR)		6 (4–10)	11.9
	UA, μmol/L, median (IQR)		398 (291–466)	5.7
	MCHC, g/L, median (IQR)		336 (329–342)	6.7
	MCV, fL, median (IQR)		88 (84–91)	9.8
	MCH, pg, median (IQR)		29 (28–30)	10.4
	MPV, fL, median (IQR)		9 (9–10)	10.4
	HCT, L/L, median (IQR)		0 (0–0)	4.7
	TC, mg/dL, median (IQR)		260 (188–388)	21.2
	TG, mg/dL, median (IQR)		162 (119–263)	25.4
	TBIL, μmol/L, median (IQR)		4 (2–6)	9.8
	DBIL, μmol/L, median (IQR)		1 (0–1)	8.8
	IBIL, μmol/L, median (IQR)		2 (1–4)	10.4
	TP, g/L, median (IQR)		47 (40–54)	4.7
	VLDL-C, mg/dL, median (IQR)		31 (19–52)	26.4
	LDL-C, mg/dL, median (IQR)		158 (110–221)	23.3
	HDL-C, mg/dL, median (IQR)		61 (50–79)	24.9
	N-HDL-C, mg/dL, median (IQR)		188 (129–293)	39.4
	APTT, second, median (IQR)		23 (20–27)	21.2
	NEU, ×10^9/L, median (IQR)		6 (4–7)	7.8
	LYM, ×10^9/L, median (IQR)		2 (1–4)	6.2
	WBC, ×10^9/L, median (IQR)		9 (7–13)	6.7
	PLT, ×10^9/L, median (IQR)		290 (231–382)	6.2
	Globulin, g/L, median (IQR)		22 (19–24)	5.2
	Albumin, g/L, median (IQR)		25 (19–31)	5.7
	A/G, median (IQR)		1 (0–1)	5.2
	Cr, μmol/L, median (IQR)		61 (37–86)	15.0
	Glucose, mmol/L, median (IQR)		4 (3–5)	33.2
	Plasma D-dimer, mg/L, median (IQR)		0 (0–0)	36.3
	Hb, g/L, median (IQR)		128 (108–144)	4.7
	LDH, U/L, median (IQR)		219 (187–278)	41.5
	CK-MB, U/L, median (IQR)		20 (13–28)	47.7
	CK, U/L, median (IQR)		41 (30–62)	47.7
	Urine WBC,/μL, median (IQR)		6 (2–14)	37.8
	Urine RBC,/μL, median (IQR)		16 (5–47)	39.9

Abbreviations: IQR, interquartile range; TDM, therapeutic drug monitoring; BMI, body mass index; PPI,; CCB, calcium channel blockers; CRP, C-reaction protein; ALT, alanine transaminase; AST, aspartate aminotransferase; PTR, prethrombin time ratio; BUN, blood urea nitrogen; UA, uric acid; MCHC, mean corpusular hemoglobin concerntration; MCV, mean corpuscular volume; MCH, mean corpuscular hemoglobin; MPV, mean platelet volume; HCT, hematocrit; TC, total cholesterol; TG, triglyceride; TBIL, total bilirubin; DBIL, direct bilirubin; IBIL, indirect bilirubin; TP, total protein; VLDL-C, very low density lipoprotein cholesterol; LDL-C, low density lipoprotein cholesterol; HDL-C, high density lipoprotein cholesterol; N-HDL-C, non-high-density lipoprotein cholesterol; APTT, activated partial thromboplastin time; LYM, lymphocyte; NEU, neutrophil; WBC, white blood cells; PLT, platelet; A/G, Albumin/Globulin; Cr, creatinine; Hb, hemoglobin; LDH, lactic dehydrogenase; CK-MB, creatine kinase-MB; CK, creatine kinase; RBC, red blood cells.

### Variable Analysis

**FIGURE 2:**
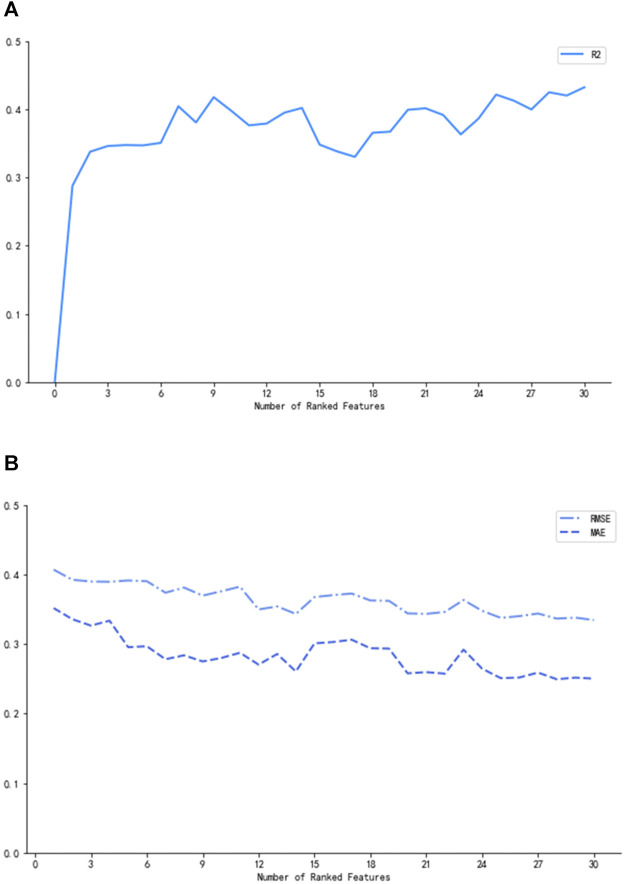
*R* ^2^ **(A)**, MAE, and RMSE **(B)** corresponding to the number of ranked variables.

In Figure 2, the three measurements reached the optimum (*R* ^2^ = 0.42, MAE = 0.27, and RMSE = 0.37) when nine of the most prominent variables were selected. Although the curves of *R* ^2^, MAE, and RMSE had slight fluctuations after the subset of nine variables, what we pursued was a concise and accurate model with minimal variables but high predictive performance. Hence, the first nine important variables were selected to establish the prediction model, including height, tacrolimus daily dose, other immunosuppressants, low-density lipoprotein cholesterol (LDL-C), mean corpuscular volume (MCV), mean corpuscular hemoglobin (MCH), white blood cell (WBC) count, direct bilirubin (DBIL), and hematocrit (HCT).

### Model Performance

**FIGURE 3:**
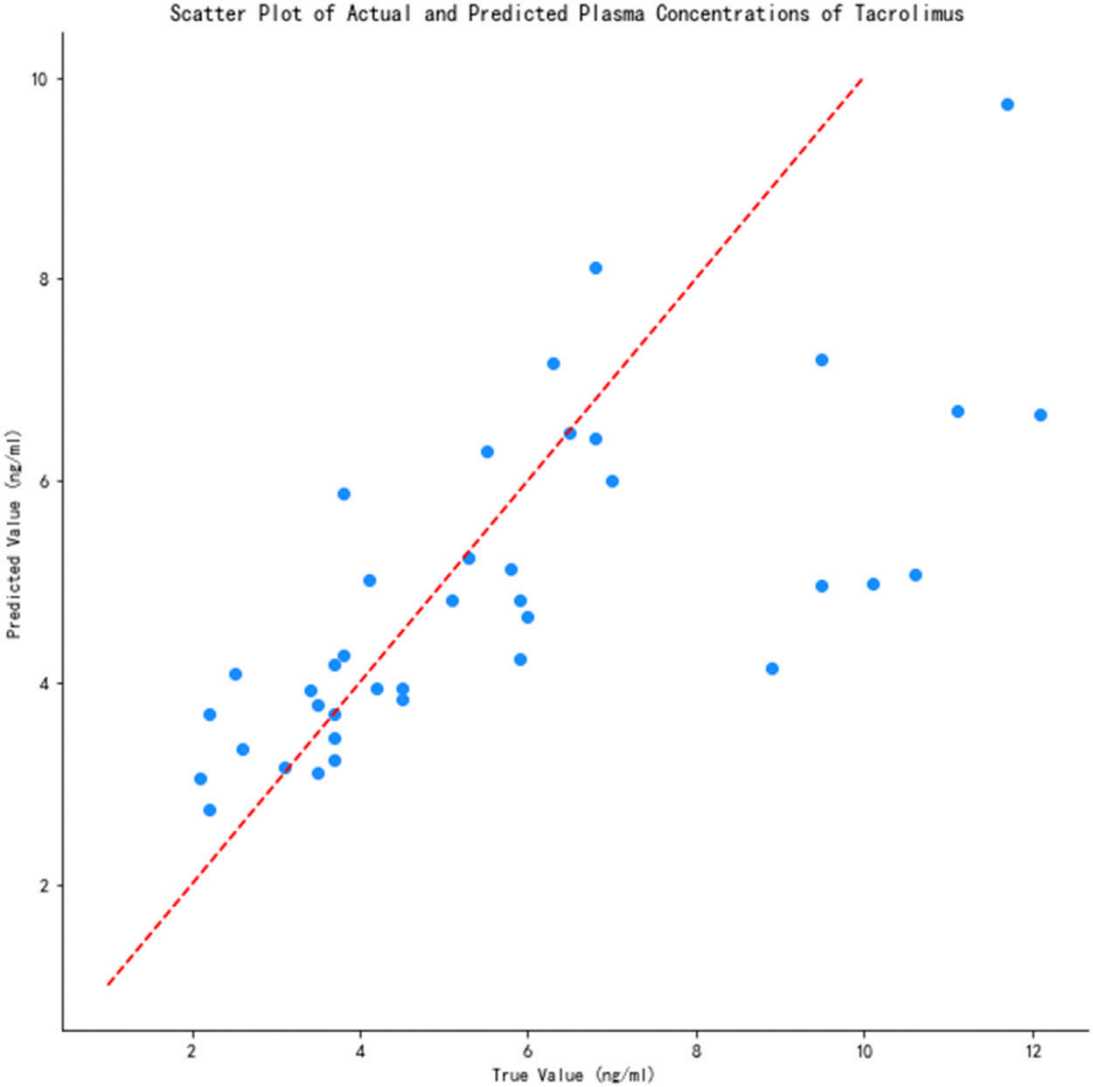
Scatter plot of actual and predicted tacrolimus TDM value.

The prediction performance of the 14 models is shown in Table 2. As can be seen, XGBoost had the highest *R* ^2^ (0.54) and lowest MAE (0.25) and RMSE (0.33) among all models. We also calculated the accuracy of the predicted concentration within ±30% of the actual concentration of the 14 models to validate the outcome, and XGBoost model gets the highest accuracy, which is 74.4%. Thus, the XGBoost model had optimal ability to predict tacrolimus TDM value. The relationship between the predicted and true values of the tacrolimus TDM value in the XGBoost model is depicted in Figure 3. The red line means predicted value equals to true value. When the blue spots get closer to the red line, the predicted results are more accurate. As illustrated, the majority of blue spots are distributed close to the red line, which can visually demonstrate the predictive accuracy for tacrolimus TDM value.

**TABLE 2:** Performance of 14 models.

Metrics	*R* ^2^	MAE	RMSE	Accuracy of the predicted concentration within ±30 (%)of the actual concentration
Models				
Liner regression	0.42	0.29	0.37	61.5
LASSO regression	0.32	0.30	0.40	48.7
Ridge regression	0.42	0.29	0.37	61.5
Elastic Net regression	0.18	0.32	0.39	53.9
Bayesian Ridge regression	0.34	0.29	0.37	56.4
KNN	0.27	0.36	0.44	51.3
SVR	0.28	0.29	0.35	53.9
Random Forest	0.45	0.29	0.38	51.3
**XGBoost**	**0.54**	**0.25**	**0.33**	**74.4**
LightGBM	0.48	0.27	0.35	61.5
CatBoost	0.36	0.30	0.36	53.6
NGBoost	0.37	0.29	0.36	56.4
AdaBoost	0.36	0.29	0.36	50.0
GradientBoosting	0.40	0.32	0.37	64.1

Abbreviations: KNN, K-nearest neighbor; SVR, Support Vector Regression; MAE, Mean Absolute Error; RMSE, Root Mean Square Error.

### Clinical Interpretation

**FIGURE 4:**
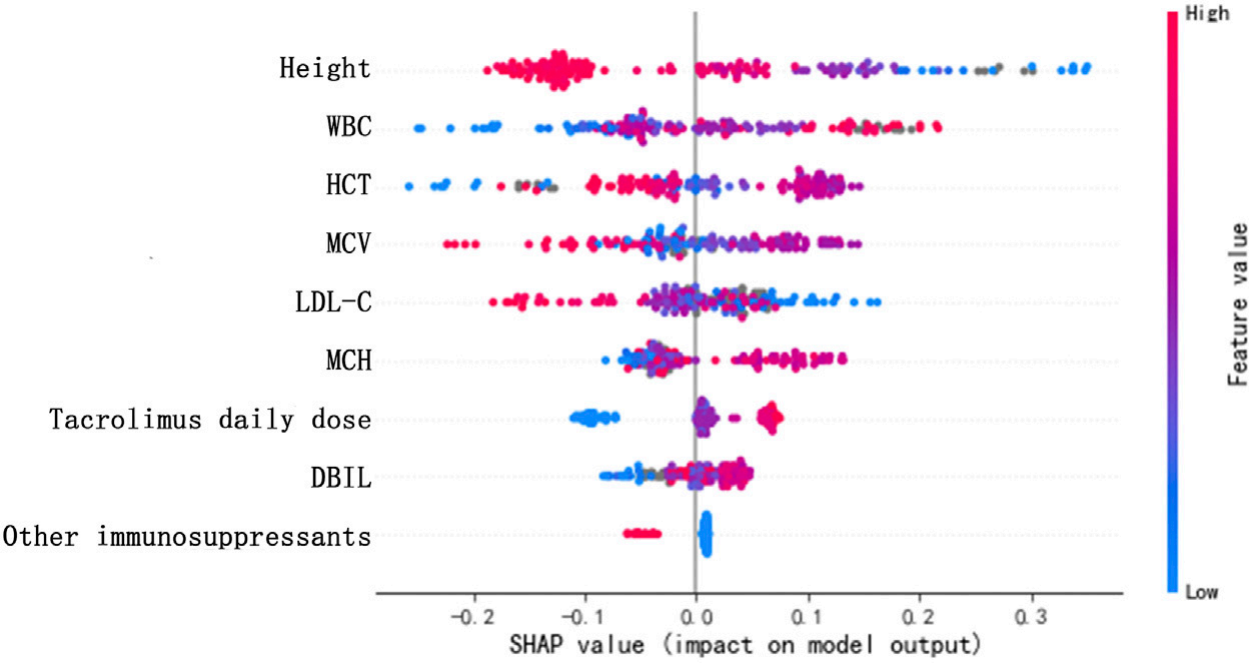
SHAP values of the important risk factors. The dot color is redder when the feature value gets higher and bluer when the feature value gets lower. When the SHAP value gets higher, the impact of the variable on model output is larger.

Table 3 shows the ranking of variable importance scores using the XGBoost model, including height, other immunosuppressants, HCT, WBC, tacrolimus daily dose, MCV, MCH, LDL-C, and DBIL, in descending order. According to the score distribution, the impacts of the nine variables on predicting tacrolimus TDM value were balanced, and the importance of height and other immunosuppressants was slightly higher than those of other variables. Furthermore, in Figure 4, SHAP values represent the impacts on model output, which is the prediction of tacrolimus TDM value. Feature value means the contribution of each variable to the predictive power of the model. For variables including tacrolimus daily dose, WBC, MCH, and DBIL, the dot color is redder when the SHAP value becomes larger, while it is bluer when the SHAP value becomes smaller, thus showing the positive impacts of these variables on tacrolimus TDM value. On the contrary, in terms of height, LDL-C, and other immunosuppressants, the dot color is bluer when the SHAP value becomes larger, while it is redder when the SHAP value becomes smaller, thus showing the negative impacts of these variables on tacrolimus TDM value. HCT and MCV did not show significant and regular positive or negative impacts on tacrolimus TDM value.

**TABLE 3:** Variable importance scores ranking.

No	Variables	Importance score
1	Height	0.1218
2	Other immunosuppressants	0.1216
3	HCT	0.1130
4	WBC	0.1120
5	Tacrolimus daily dose	0.1112
6	MCV	0.1105
7	MCH	0.1061
8	LDL-C	0.1034
9	DBIL	0.1004

Abbreviations: TDM, therapeutic drug monitoring; HCT, hematocrit; WBC, white blood cells; MCV, mean corpuscular volume; MCH, mean corpuscular hemoglobin; LDL-C, low density lipoprotein cholesterol; DBIL, direct bilirubin.

## Discussion

In this study, a machine learning model was established to predict the blood drug concentration of tacrolimus in patients with autoimmune diseases. A feature SFS algorithm was applied to screen model variables, and five linear models and nine machine learning models were used for comparison. Ultimately, the XGBoost algorithm was selected to build the prediction model with an *R* ^2^ of 0.54, which achieved a high prediction accuracy in the absence of genetic information. This finding indicated that the XGBoost model has good predictive ability and can be used to predict the blood concentration of patients after taking tacrolimus.

Among the selected important variables in the prediction model, some were identified by previous studies, and some were newfound influencing variables. For instance, height was rarely found to have a relationship with tacrolimus concentration in other studies, whereas it had high importance in our prediction model (importance score = 0.1218). Only one population pharmacokinetic model in Asian liver transplant patients mentioned that height influenced the apparent volume of tacrolimus distribution on the basis of whole blood concentrations after oral administration. (Sam et al., 2006) In our study, the sample age distribution was relatively discrete. Among the 193 cases, 100 individuals were aged below 18 years, and their height data were greatly influenced by individual variability. Hence, height was important in the present model. However, future prediction models could be established according to age stratification to avoid bias.

Other immunosuppressants, including ciclosporin, mycophenolate mofetil, cyclophosphamide, azathioprine, and methotrexate, were identified as important variables with negative impacts on tacrolimus concentration. Previous studies mostly discussed the comparison of the therapeutic effects of these drugs. Few investigated the impacts of other immunosuppressants on tacrolimus concentration. As calcineurin inhibitors, the main effect of tacrolimus and ciclosporin was the inhibition of T-cell function. (Schreiber and Crabtree, 1992) Combination drug therapy with ciclosporin may reduce the absorption of tacrolimus in the blood, leading to low concentration. Similarly, other immunosuppressants may influence tacrolimus concentration due to combined therapy. However, data are lacking to explain this relationship, and more research is needed in the future.

The most commonly discussed variable influencing tacrolimus concentration was HCT. Several studies on transplant recipients have identified HCT to affect tacrolimus clearance. (Undre and Schäfer, 1998; Lee et al., 2006; Brunet et al., 2019) To be more specific, Staatz C, et al. and Sam W, et al. have quantified the impact of HCT on tacrolimus apparent clearance; they found that an increase in tacrolimus clearance was associated with decreased HCT levels. (Sam et al., 2000; Staatz et al., 2002) Delays in drug clearance indicate the accumulation of tacrolimus in the body, leading to higher concentration. In addition, low level of HCT may reduce the proportion of tacrolimus bound to red blood cells and increase the proportion bound to plasma, which can be more easily metabolized by the liver. (Li et al., 2011; Brunet et al., 2019) Therefore, low HCT level could result in a decrease in total tacrolimus concentration in the whole blood. This could also interpret the relationship between MCV and tacrolimus blood concentration. Nevertheless, the specific quantified impacts of HCT and MCV on tacrolimus blood concentration in our study were unclear. Larger samples may be needed for investigation in the future. Regarding MCH, low HCT level may lead to low MCH value. Moreover, it was positively associated with tacrolimus concentration in the present study.

A study on Chinese renal transplant recipients taking tacrolimus as immunosuppressive drug found that total bilirubin was a significant influencing factor on maintenance tacrolimus dose. (Li et al., 2011) Our results indicate that DBIL was significantly related to tacrolimus concentration and showed a reverse association. According to the findings of Bellarosa, et al., bilirubin and tacrolimus competitively bind with P-glycoprotein; thus, high bilirubin level may delay the transport and metabolism of tacrolimus, resulting in high blood concentration. (Bellarosa et al., 2009) Additionally, high bilirubin level indicates impaired liver function and poor liver metabolism, which may lead to increased tacrolimus concentration in the blood.

In terms of other influencing variables, daily dose shows obvious positive relationship with tacrolimus concentration, illustrating that using high dose of tacrolimus daily could contribute to high tacrolimus blood concentration. WBC was positively related to tacrolimus concentration, and LDL-C was negatively related to tacrolimus concentration, both of which were rarely investigated in previous studies. Increased WBC counts imply that the body is attacked by an infection, requiring intensive drug treatment. Thus, the use of tacrolimus is probably increased, leading to higher blood concentrations. High level of LDL-C could represent liver dysfunction and was theoretically associated with high blood concentration of tacrolimus. However, our study shows the opposite findings. These results should be validated in the future with larger sample size and higher quality data. Previous studies found that some factors, including CYP3A5 genotype, bodyweight, liver and renal function, serum albumin, and coadministration of diltiazem and fluconazole, are related to the concentration, dose, and clearance of tacrolimus; however, they were not significant in our model. (Zahir et al., 2005; Sam et al., 2006; Li et al., 2011; Brunet et al., 2019) In this study, genetic data were too scarce to be used for exploration, and further research should include more factors in the future.

Most previous research established pharmacokinetic models. In real-world studies, variables are not always independent to each other, and the majority have closely non-linear relationships. According to the second consensus report about TDM of tacrolimus-personalized therapy issued by the Immunosuppressive Drugs Scientific Committee of the International Association of Therapeutic Drug Monitoring and Clinical Toxicity in 2017, *R* ^2^ values have great fluctuation with a range from 0.27 to 0.99 in the prediction of tacrolimus concentration using pharmacokinetic models. (Brunet et al., 2019) Our prediction model using XGBoost algorithm achieved an *R* ^2^ of 0.54 and the lowest MAE and RMSE, which outperformed other models investigated herein. One study used the SVR model to predict tacrolimus blood concentration in liver transplantation patients; the authors found that SVR models show superior prediction accuracy than multiple linear regression model. (Van Looy et al., 2007) Compared with pharmacokinetic models and linear regression models, machine learning models can capture the complex relationships of variables and analyze high-dimension data in clinical practice. Considering that our data were derived from real-world settings with some missing values, machine learning models have better ability to deal with incomplete data.

In conclusion, on the basis of 193 cases of tacrolimus TDM data from real-world settings, the study identified nine important variables for tacrolimus concentration, including height, other immunosuppressants, HCT, WBC, tacrolimus daily dose, MCV, MCH, LDL-C, and DBIL, and their specific quantified impacts. Then, we established a prediction model of tacrolimus concentration in patients with autoimmune disease *via* XGBoost algorithm, which had better prediction abilities than other models compared in the study.

To our knowledge, this study is the first to predict the blood concentration of tacrolimus in patients with autoimmune diseases using machine learning techniques on the basis of RWE. The prediction model is of important clinical significance as regimen adjustment may be required to maintain blood concentrations within the therapeutic range in patients whose clinical data are changing. One limitation is the limited sample size, and all data were obtained from one center. In the future, larger data from multiple centers are needed for further analysis.

## Data Availability

The raw data supporting the conclusions of this article will be made available by the authors, without undue reservation.

## References

[B1] Aha D. W. Kibler D. Albert M. K. (1991). Instance-based Learning Algorithms. Mach. Learn. 6, 37–66. 10.1007/bf00153759

[B2] Andrews L. M. Li Y. De Winter B. C. M. Shi Y. Y. Baan C. C. Van Gelder T. (2017). Pharmacokinetic Considerations Related to Therapeutic Drug Monitoring of Tacrolimus in Kidney Transplant Patients. Expert Opin. Drug Metab. Toxicol. 13 (12), 1225–1236. 10.1080/17425255.2017.1395413 29084469

[B3] Bellarosa C. Bortolussi G. Tiribelli C. (2009). The Role of ABC Transporters in Protecting Cells from Bilirubin Toxicity. Curr. Pharm. Des. 15 (25), 2884–2892. 10.2174/138161209789058246 19754365

[B4] Breiman L. (2001). Random Forests. Mach. Learn. 45, 5–32. 10.1023/a:1010933404324

[B5] Brunet M. van Gelder T. Åsberg A. Haufroid V. Hesselink D. A. Langman L. (2019). Therapeutic Drug Monitoring of Tacrolimus-Personalized Therapy: Second Consensus Report. Ther. Drug Monit. 41 (3), 261–307. 10.1097/FTD.0000000000000640 31045868

[B6] Chen H. X. Cheng Q. Li F. He Q. N. Cao Y. Yi Z. W. (2020). Efficacy and Safety of Tacrolimus and Low-Dose Prednisone in Chinese Children with Steroid-Resistant Nephrotic Syndrome. World J. Pediatr. 16 (2), 159–167. 10.1007/s12519-019-00257-z 31049814

[B7] Chen T. Guestrin C. (2016). XGBoost. ACM. 10.1145/2939672.2939785

[B8] Cremers S. Guha N. Shine B. (2016). Therapeutic Drug Monitoring in the Era of Precision Medicine: Opportunities!. Br. J. Clin. Pharmacol. 82 (4), 900–902. 10.1111/bcp.13047 27612297 PMC5137816

[B9] Hannah J. Casian A. D'Cruz D. (2016). Tacrolimus Use in Lupus Nephritis: A Systematic Review and Meta-Analysis. Autoimmun. Rev. 15 (1), 93–101. 10.1016/j.autrev.2015.09.006 26427983

[B10] Hao G. X. Huang X. Zhang D. F. Zheng Y. Shi H. Y. Li Y. (2018). Population Pharmacokinetics of Tacrolimus in Children with Nephrotic Syndrome. Br. J. Clin. Pharmacol. 84 (8), 1748–1756. 10.1111/bcp.13605 29637588 PMC6046506

[B11] Huang L. Wang J. Yang J. Zhang H. Ni Y. Zhu Z. (2019). Impact of CYP3A4/5 and ABCB1 Polymorphisms on Tacrolimus Exposure and Response in Pediatric Primary Nephrotic Syndrome. Pharmacogenomics 20 (15), 1071–1083. 10.2217/pgs-2019-0090 31588879

[B12] Johnston A. (2013). Equivalence and Interchangeability of Narrow Therapeutic index Drugs in Organ Transplantation. Eur. J. Hosp. Pharm. 20 (5), 302–307. 10.1136/ejhpharm-2012-000258 24089632 PMC3786630

[B13] Ke G. Meng Q. Finley T. Wang T. Chen W. Ma W. (2017). “A Highly Efficient Gradient Boosting Decision Tree.” in Proceedings of the Advances in Neural Information Processing Systems. (Long Beach, CA, USA: Curran Associates, Inc.), 3146–3154.

[B14] Lee J. Y. Hahn H. J. Son I. J. Suh K. S. Yi N. J. Oh J. M. (2006). Factors Affecting the Apparent Clearance of Tacrolimus in Korean Adult Liver Transplant Recipients. Pharmacotherapy 26 (8), 1069–1077. 10.1592/phco.26.8.1069 16863483

[B15] Li L. Li C. J. Zheng L. Zhang Y. J. Jiang H. X. Si-Tu B. (2011). Tacrolimus Dosing in Chinese Renal Transplant Recipients: a Population-Based Pharmacogenetics Study. Eur. J. Clin. Pharmacol. 67 (8), 787–795. 10.1007/s00228-011-1010-y 21331500

[B16] Lundberg S. M. Lee S. I. (2017). “A Unified Approach to Interpreting Model Predictions.” in Advances in Neural Information Processing Systems. Long Beach, CA: Neural Information Processing Systems, 4765–4774. Available at: https://github.com/slundberg/shap

[B17] Miyasaka N. Kawai S. Hashimoto H. (2009). Efficacy and Safety of Tacrolimus for Lupus Nephritis: a Placebo-Controlled Double-Blind Multicenter Study. Mod. Rheumatol. 19 (6), 606–615. 10.1007/s10165-009-0218-5 19688181

[B18] Mok C. C. (2017). Calcineurin Inhibitors in Systemic Lupus Erythematosus. Best Pract. Res. Clin. Rheumatol. 31 (3), 429–438. 10.1016/j.berh.2017.09.010 29224682

[B19] Mok C. C. (2017). Therapeutic Monitoring of the Immuno-Modulating Drugs in Systemic Lupus Erythematosus. Expert Rev. Clin. Immunol. 13 (1), 35–41. 10.1080/1744666X.2016.1212659 27417340

[B20] Prokhorenkova L. Gusev G. Vorobev A. Dorogush A. V. Gulin A. (2017). Catboost: Unbiased Boosting with Categorical Features. arXiv Preprint arXiv:1706.09516.

[B21] Sam W. J. Aw M. Quak S. H. Lim S. M. Charles B. G. Chan S. Y. (2000). Population Pharmacokinetics of Tacrolimus in Asian Paediatric Liver Transplant Patients. Br. J. Clin. Pharmacol. 50 (6), 531–541. 10.1046/j.1365-2125.2000.00288.x 11136292 PMC2015016

[B22] Sam W. J. Tham L. S. Holmes M. J. Aw M. Quak S. H. Lee K. H. (2006). Population Pharmacokinetics of Tacrolimus in Whole Blood and Plasma in Asian Liver Transplant Patients. Clin. Pharmacokinet. 45 (1), 59–75. 10.2165/00003088-200645010-00004 16430311

[B23] Schreiber S. L. Crabtree G. R. (1992). The Mechanism of Action of Cyclosporin A and FK506. Immunol. Today 13 (4), 136–142. 10.1016/0167-5699(92)90111-J 1374612

[B24] Staatz C. E. Willis C. Taylor P. J. Tett S. E. (2002). Population Pharmacokinetics of Tacrolimus in Adult Kidney Transplant Recipients. Clin. Pharmacol. Ther. 72 (6), 660–669. 10.1067/mcp.2002.129304 12496747

[B25] Undre N. A. Schäfer A. (1998). Factors Affecting the Pharmacokinetics of Tacrolimus in the First Year after Renal Transplantation. European Tacrolimus Multicentre Renal Study Group. Transpl. Proc 30 (4), 1261–1263. 10.1016/s0041-1345(98)00234-6 9636512

[B26] Van Looy S. Verplancke T. Benoit D. Hoste E. Van Maele G. De Turck F. (2007). A Novel Approach for Prediction of Tacrolimus Blood Concentration in Liver Transplantation Patients in the Intensive Care Unit through Support Vector Regression. Crit. Care 11 (4), R83. 10.1186/cc6081 17655766 PMC2206504

[B27] Xu Z. Wang Z. (2019). A Risk Prediction Model for Type 2 Diabetes Based on Weighted Feature Selection of Random Forest and XGBoost Ensemble Classifier. 2019 Eleventh Int. Conf. Adv. Comput. Intelligence (Icaci). 10.1109/icaci.2019.8778622

[B28] Zahir H. McLachlan A. J. Nelson A. McCaughan G. Gleeson M. Akhlaghi F. (2005). Population Pharmacokinetic Estimation of Tacrolimus Apparent Clearance in Adult Liver Transplant Recipients. Ther. Drug Monit. 27 (4), 422–430. 10.1097/01.ftd.0000170029.36573.a0 16044097

[B29] Zhang Y. Zhang R. (2018). Recent Advances in Analytical Methods for the Therapeutic Drug Monitoring of Immunosuppressive Drugs. Drug Test. Anal. 10 (1), 81–94. 10.1002/dta.2290 28851030

